# Association Between Triglyceride-Glucose Index, Blood Pressure Status, and Coronary Heart Disease Risk Among Chinese Adults With Disabilities: 10-Year Disability Health Survey Cohort Study

**DOI:** 10.2196/78068

**Published:** 2025-11-03

**Authors:** Hui Liu, Yao Li, Yiyan Wang, Tao Wang, Chenghua Jiang, Hengjing Wu, Jing Wu

**Affiliations:** 1 Shanghai YangZhi Rehabilitation Hospital (Shanghai Sunshine Rehabilitation Center), School of Medicine, Tongji University, Guangxing Road 2209, Shanghai, 201619, China, 86 13817556859; 2 School of Nursing, Wannan Medical College, Wuhu, China

**Keywords:** disabilities, coronary heart disease, CHD, risk, triglyceride-glucose index, blood pressure

## Abstract

**Background:**

The triglyceride-glucose (TyG) index and blood pressure (BP) status are key indicators associated with coronary heart disease (CHD). However, limited research has focused on individuals with disabilities.

**Objective:**

This study explores the potential combined effects of the TyG index and BP status on CHD risk in groups with varying disability characteristics.

**Methods:**

This study analyzed data from the Shanghai Disability Health Survey, conducted between January 2012 and December 2022. Participants were then categorized into 3 BP status groups: nonelevated BP, elevated BP, and hypertension. Cox proportional hazards regression models were used to assess the associations between BP status, the TyG index, and CHD incidence. Additionally, the mediating, interaction, and combined effects of these factors on CHD risk were examined. A stratified analysis was performed based on participants’ disability characteristics, including disability type and severity, to explore potential variations in the associations.

**Results:**

Among the 21,628 participants, the mean age was 53.30 (SD 10.57) years, and 50.89% (11007/21628) were male. In a follow-up of 77.45 months, CHD events occurred in 2312 participants (10.69%). The TyG index and BP status were independently associated with an increased risk of CHD. Mediation analysis showed that TyG explained 20.5% (95% CI 13.6%‐22.0%) of the BP and CHD association. Significant multiplicative interactions were identified (hazard ratio [HR] 1.41, 95% CI 1.02‐1.94), and joint analysis indicated the highest CHD risk in those with both hypertension and elevated TyG (HR 1.92, 95% CI 1.52‐2.42). Stratified analyses revealed stronger mediation in participants with physical disabilities (22.6%, 95% CI 9.0%‐60%) or visual disabilities (16.6%, 95% CI 4.8%‐51%), while this was not significant in those with hearing or speech (*P*=.07) or intellectual or mental disabilities (*P*=.13). By disability grading, the mediated proportion was 22.3% (95% CI 9.2%‐59.4%) in mild or moderate and 18.8% (95% CI 15.7%‐29%) in severe or very severe groups. Joint associations showed consistently higher CHD risk across most disability classifications, with particularly elevated risk in people with intellectual or mental disabilities (HR 3.51, 95% CI 1.89‐6.50).

**Conclusions:**

BP and the TyG index were significantly associated with CHD risk in individuals with disabilities, with TyG mediating a part of this association and showing stronger effects in physical and mild to moderate disabilities. Significant interactions between BP and TyG further highlight their combined impact, underscoring the need for integrated interventions targeting both factors.

## Introduction

### Background

Coronary heart disease (CHD) is a leading cause of cardiovascular mortality worldwide, with particularly high prevalence in certain high-risk populations [1,2]. While there is extensive research on CHD in the general population [3,4], studies on CHD in individuals with disabilities remain scarce, despite the fact that this group constitutes a significant portion of the global population. According to the latest report by the World Health Organization, over 1 billion people (approximately 15% of the global population) live with some form of disability [5]. Individuals with disabilities are at an elevated risk for cardiovascular diseases (CVDs) due to functional limitations, metabolic disorders, and restricted physical activity [6,7]. Hypertension is a recognized risk factor for CHD, closely linked to atherosclerosis, left ventricular hypertrophy, and other detrimental cardiovascular outcomes. Among people with disabilities, the prevalence of hypertension is often higher, further exacerbating the cardiovascular burden in this population [8].

Recently, the triglyceride-glucose (TyG) index has gained widespread application as a biomarker of insulin resistance in assessing CVD risk and adverse outcomes [9]. Derived from the product of fasting glucose and triglycerides, the TyG index provides an indirect measure of insulin sensitivity. Studies show that the TyG index independently and significantly predicts several CVDs and poor prognoses [10,11], with a higher TyG index being significantly associated with increased risks of stroke [12], heart failure [13], and all-cause mortality [14]. Moreover, interactions between the TyG index and other common cardiovascular risk factors may yield additional impacts on cardiovascular risk. For example, research has shown that the TyG index mediates a substantial portion of the association between BMI and stroke [12], and a co-exposure and mutual mediation effect has been observed between the TyG index and high-sensitivity C-reactive protein for CVD [15]. These findings further support the potential of the TyG index as a tool for predicting CVD risk.

Given that both blood pressure (BP) and the TyG index are closely associated with CHD risk, their potential combined effect on CVD has garnered growing attention. Multiple studies indicate that hypertension and insulin resistance may interact, and their joint evaluation and intervention may have important implications for CVD prevention [16,17]. A more recent cohort study of 57,192 participants using cross-lagged analysis found that elevated TyG exerted a stronger impact on subsequent BP changes than the reverse effect [18]. However, there is still a lack of research focused on systematically evaluating the combined effects of BP and the TyG index on CHD, particularly their impact among people with disabilities.

### Objectives

Based on a 10-year longitudinal cohort of people with disabilities, we aim to evaluate the independent and combined effects of different BP levels and the TyG index on incident CHD in people with disabilities, as well as to further analyze the potential mediating role of the TyG index in the association between BP and CHD.

## Methods

### Data Collection

This study is a retrospective cohort data analysis based on information sourced from the Shanghai Disabled Persons Rehabilitation Comprehensive Information Platform, established by the Shanghai Disabled Persons’ Federation. Since 2012, the Shanghai Disabled Persons’ Federation has provided annual free health screening services for local individuals with disabilities. As of December 2022, the Shanghai Sunshine Rehabilitation Center (Shanghai Sunshine Rehabilitation Hospital) has facilitated screenings for more than 20,000 persons with disabilities, and the health data obtained have been uploaded to the Shanghai Disabled Persons Rehabilitation Comprehensive Information Platform for health monitoring and scientific research purposes. The recruitment process for this study was based on participants attending the annual health screenings, where individuals with various types of disabilities voluntarily participated. The recruitment aimed to include a diverse range of disability types and severities.

### Ethical Considerations

This study was approved by the Ethics Committee of Shanghai Sunshine Rehabilitation Hospital (2019‐051). All participants were informed that their health screening data could be used for scientific research, and informed consent was obtained prior to data collection. To ensure privacy and confidentiality, all data used in this analysis were anonymized and deidentified before being accessed by the research team. Participants did not receive financial compensation for their participation. No individual participants are identifiable in any images or supplementary materials associated with this study.

### Study Population

The study population included individuals with disabilities meeting the following criteria: (1) aged over 18 years and (2) participated in at least 2 health screenings during the 10-year follow-up period from 2012 to 2022, with complete electronic health records for each screening. Exclusion criteria were as follows: (1) a prior diagnosis of CHD at baseline, confirmed by a qualified physician in accordance with the World Health Organization diagnostic criteria; (2) presence of other severe conditions, such as malignancies, organ failure, or major trauma; (3) women who were pregnant, breastfeeding, or planning to become pregnant; and (4) individuals with multiple types of disabilities rather than a single disability type. The final eligible sample consisted of 21,628 individuals (Figure S1 in Multimedia Appendix 1).

### Assessment of BP and TyG

After participants rested for approximately 10 minutes, trained nursing staff measured BP using a standardized procedure with an automated arm BP monitor (Omron Corp). BP was recorded twice. For participants with upper limb amputation or injuries that could lead to measurement errors, physicians conducted supine BP measurements on both ankles via the posterior tibial artery and recorded the reading from the limb with the higher value. To ensure consistency with standard arm BP readings, leg BP values were corrected following recommendations in references [18].

BP status was classified based on the 2024 Chinese Hypertension Prevention and Treatment Guidelines [19]. Participants were categorized into 3 groups: nonelevated BP, elevated BP, and hypertension. Nonelevated BP was defined as systolic BP (SBP) <120 mm Hg and diastolic BP (DBP) <80 mm Hg, without a history of hypertension. Elevated BP was defined as SBP ranging from 120 mm Hg to 139 mm Hg or DBP from 80 mm Hg to 89 mm Hg. Hypertension was defined as SBP≥140 mm Hg, DBP≥90 mm Hg, or a self-reported history of hypertension.

The TyG index, used as a marker of metabolic health, was calculated from venous blood samples. All blood samples were immediately frozen after collection and transported to a specialized laboratory, where they were stored at –80°C to maintain sample quality. Serum triglyceride (TG) and fasting plasma glucose (FPG) levels were measured using an enzymatic colorimetric method, and the TyG index was calculated according to the formula: ln [TG (mg/dL)×FPG (mg/dL)/2] [20,21]. Both the TyG index and BP were measured at baseline for the analysis.

### Outcome Ascertainment

The primary outcome of this study was the incidence of CHD occurring from the time of health screening in 2012 until December 2022. CHD was defined as fatal ischemic heart disease (according to ICD-10 codes I20-I25) or nonfatal myocardial infarction (ICD-10 codes I21-I23). The CHD history was assessed through interviews with qualified physicians and verified by reviewing patient medical records. The timing of CHD onset was recorded based on case documentation to ensure the reliability and temporal accuracy of outcome event data.

### Covariates

Participants’ medical and functional status was comprehensively evaluated by trained health care professionals at baseline. The health questionnaire collected data on general demographics (eg, gender, age, and marital status); medical history (eg, hypertension, diabetes, and fatty liver); and disability status (classification and severity). The classification and grading of disabilities were based on China’s National Standard “Classification and Grading Criteria of Disabilities” (GB/T 26341‐2010) [22]. Hospital health examinations included anthropometric measurements (eg, height and weight); biochemical assessments (eg, complete blood count, blood biochemistry, and urinalysis); and electrocardiogram and ultrasound examinations. Blood tests were conducted after an overnight fast of at least 12 hours.

### Statistical Analyses

In the statistical analysis, continuous variables that follow a normal distribution were expressed as means and SDs, while nonnormally distributed data were reported as medians and IQR. Categorical variables were presented as frequencies and percentages. For comparisons of baseline characteristics across groups based on BP (divided into 3 categories), chi-square tests, ANOVA, or Kruskal-Wallis rank-sum tests were used according to the data type. Missing data were handled using multiple imputation with chained equations using the *MICE* package of R (version 4.3.0; R Foundation for Statistical Computing) to minimize bias. Detailed information on the missing data can be found in Table S1 in Multimedia Appendix 1.

To estimate the association between different BP categories (nonelevated BP, elevated BP, and hypertension); TyG quartiles; and outcomes, we used Cox proportional hazards regression models to calculate hazard ratios (HRs) and 95% CIs. The models were adjusted in 3 stages: model 1 adjusted for age and gender; model 2 further adjusted for disability-specific factors—disability type and severity; and model 3 additionally controlled for marital status, education level, diabetes history, and serum creatinine level. Furthermore, restricted cubic spline (RCS) models with 3 knots were used to explore nonlinear associations between TyG and the outcome.

To assess the mediating effect of TyG between BP and CHD, we used the *mediation* package in R. First, a logistic regression model (mediator model) was built with the independent and confounding variables, using the mediator variable as the outcome. A second Cox proportional hazards regression model (outcome model) included the independent variable and mediator, with CHD as the outcome. This approach allowed us to estimate both the indirect and direct effects, showing the proportion of the total effect mediated by TyG. The proportion mediated was calculated as the ratio of the natural indirect effect (NIE) to the sum of the natural direct effect and NIE. The bootstrap method was used to estimate the 95% CI of the indirect effect. TyG was dichotomized based on an RCS-determined threshold (8.56). In R, the *mets* package was used to calculate BP’s NIE and natural direct effect on CHD incidence through TyG as a mediator.

To further explore the association between TyG and CHD, stratified analyses by BP level were conducted. Interaction terms between BP (nonelevated BP, elevated BP, and hypertension) and TyG quartiles were included, and a likelihood ratio test was performed to compare models with and without interaction terms, assessing whether interaction improved model fit [23]. For joint association analyses, participants were categorized into 12 groups based on their BP and TyG quartiles, with HRs for CHD calculated and compared against a reference group with nonelevated BP and TyG in the first quartile.

Given the unique characteristics of individuals with disabilities, subgroup analyses were performed by disability type and severity. Participants were divided into those with limb disabilities and other disability types, as well as into mild and moderate versus severe and very severe categories. Primary analyses, including mediation analysis, stratified mediation, interaction, and joint association analyses, were repeated within these subgroups.

## Results

### Baseline Characteristics

A total of 21,628 eligible participants were included in the study, with details on the sample selection process illustrated in Figure S1 in Multimedia Appendix 1. The mean age of the participants was 53.3 (SD 10.57) years, and 10,621 (49.11%) were female. More than half of the participants (11,901/21,628, 55.00%) had physical disabilities, with the majority classified as having either mild or severe disabilities (16,975/21,628, 78.49%). The incidence rate per year is provided in Table S2 in Multimedia Appendix 1.

RCS regression was used to explore the potential nonlinear associations between TyG and CHD events. The results revealed a nonlinear association between TyG and the risk of CHD (*P*=.002; Figure S2A and S2B in Multimedia Appendix 1). Therefore, nonlinear modeling was adopted to better capture the association between TyG and CHD risk. BP categories were based on the 2024 “Chinese Guidelines for the Prevention and Treatment of Hypertension,” while TyG was classified according to the commonly used quartile method in the literature.

Baseline characteristics of the participants, categorized by baseline BP into nonelevated BP (4964/21628, 22.95%), elevated BP (1321/21628, 54.28%), and hypertension (610/21628, 22.77%) groups, are compared in Table 1. The average TyG index at baseline was 8.62 (SD 0.60). Table S3 in Multimedia Appendix 1 provides the baseline characteristics of participants based on TyG quartiles.

**Table 1.** Baseline characteristics of 21,628 participants with disabilities by blood pressure (BP) level (N=21,628).

Variables	Total sample	Nonelevated BP (SBP^a^<120, DBP^b^<80; n=4964)	Elevated BP (120≤SBP<140, 80≤DBP<90; n=11,739)	Hypertension (SBP≥140, DBP≥90; n=4925)	*P* value^c^
Age (y), mean (SD)	53.30 (10.57)	49.62 (11.03)	53.75 (10.36)	55.93 (9.54)	<.001
Sex, n (%)					<.001
Male	11,007 (50.89)	2325 (46.84)	6064 (51.66)	2618 (53.16)	
Female	10,621 (49.11)	2639 (53.16)	5675 (48.34)	2307 (46.84)	
BMI (kg/m^2^), mean (SD)	24.09 (3.58)	22.40 (3.09)	24.27 (3.45)	25.37 (3.69)	<.001
Marital status, n (%)					<.001
Married	17,852 (82.54)	3874 (78.04)	9788 (83.38)	4190 (85.08)	
Other	3776 (14.46)	1090 (21.96)	1951 (16.62)	735 (14.92)	
Education, n (%)					<.001
Primary school and illiterate	4683 (21.70)	1033 (20.81)	2535 (21.59)	1115 (22.64)	
Junior high school	11,199 (51.80)	2596 (52.30)	6092 (51.90)	2511 (50.98)	
Senior high school	4896 (22.60)	1129 (22.74)	2671 (22.75)	1096 (22.25)	
College and higher	850 (3.93)	206 (4.15)	441 (3.76)	203 (4.12)	
Classification of disabilities, n (%)					<.001
Intellectual and mental disability	3799 (17.60)	1047 (21.09)	1989 (16.94)	763 (15.49)	
Hearing and speech disability	1775 (8.21)	476 (9.59)	908 (7.73)	391 (7.94)	
Visual disability	4153 (19.20)	900 (18.13)	2302 (19.61)	951 (19.31)	
Physical disability	11,901 (55.00)	2541 (51.19)	6540 (55.71)	2820 (57.26)	
Grading of disabilities, n (%)					<.001
Very severe	1924 (8.90)	485 (9.77)	996 (8.48)	443 (8.99)	
Severe	2729 (12.60)	1689 (34.02)	4095 (34.88)	1674 (33.99)	
Moderate	7458 (34.50)	692 (13.94)	1453 (12.38)	584 (11.86)	
Mild	9517 (44.00)	2098 (42.26)	5195 (44.25)	2224 (45.16)	
Comorbidities, n (%)					<.001
Hypertension	5961 (27.60)	281 (5.66)	3125 (26.62)	2555 (51.88)	
Diabetes	1317 (6.09)	174 (3.35)	459 (5.75)	684 (8.08)	
Blood pressure, mean (SD)					<.001
SBP	135.15 (20.87)	109.39 (7.47)	135.78 (12.28)	159.61 (15.13)	
DBP	79.31 (12.46)	65.56 (7.05)	79.70 (7.40)	92.25 (11.94)	
Metabolic biomarkers					
FBG^d^ (mmol/L), mean (SD)	5.60 (1.53)	5.20 (1.11)	5.62 (1.52)	5.94 (1.82)	<.001
TC^e^ (mmol/L), mean (SD)	4.58 (0.87)	4.37 (0.77)	4.79 (0.90)	4.94 (0.93)	<.001
TG^f^ (mmol/L), median (IQR)	1.22 (0.8-1.75)	1.02 (0.76-1.43)	1.24 (0.89-1.77)	1.41 (1.00-2.02)	<.001
TP^g^ (g/L), mean (SD)	72.38 (3.61)	71.44 (4.12)	72.40 (4.12)	73.29 (4.25)	<.001
Alb^h^ (g/L), mean (SD)	43.43 (2.33)	43.16 (2.35)	43.46 (2.31)	43.62 (2.38)	<.001
Glo^i^(g/L), mean (SD)	28.97 (3.61)	28.37 (3.65)	28.96 (3.52)	29.60 (3.68)	<.001
Alt^j^ (U/L), median (IQR)	20.00 (15.00-29.00)	18.00 (13.00-25.00)	21.00 (15.00-29.00)	22.00 (16.00-32.00)	.115
UA^k^ (μmol/L), mean (SD)	318.40 (87.54)	299.65 (81.11)	319.51 (86.62)	334.67 (92.29)	<.001
SCr^l^ (μmol/L), median (IQR)	62.10 (50.70-73.32)	61.20 (50.10-72.50)	62.20 (50.70-73.40)	62.80 (51.50-74.10)	<.001
SU^m^ (mmol/L), median (IQR)	5.00 (4.20-6.00)	4.90 (4.10-5.80)	5.10 (4.30-6.00)	5.10 (4.30-6.00)	<.001
Hemoglobin (g/L), mean (SD)	137.01 (15.27)	133.80 (15.31)	137.39 (15.08)	139.38 (15.16)	<.001
Platelet count (10^9/L), mean (SD)	200.88 (58.27)	198.63 (57.69)	200.63 (57.81)	203.73 (59.82)	<.001
TyG^n^	8.62±0.60	8.38±0.54	8.64±0.59	8.80±0.62	<.001

a SBP: systolic blood pressure.

b DBP: diastolic blood pressure.

c The *P *value was determined using the chi-square test for categorical data, ANOVA for continuous data across categorical groups, and the Kruskal-Wallis test for continuous data that are ordinal or nonnormally distributed.

d FBG: fasting plasma glucose.

e TC: total cholesterol.

f TG: total triglyceride.

g TP: total protein.

h Alb: albumin

i Glo: globulin

j Alt: alanine aminotransferase.

k UA: uric acid.

l SCr: serum creatinine.

m SU: serum urea.

n TyG: triglyceride-glucose. The TyG was calculated using the formula ln [TC (mg/dl)×FBG (mg/dl)/2].

### Association of TyG and BP With CHD

**Figure 1.**
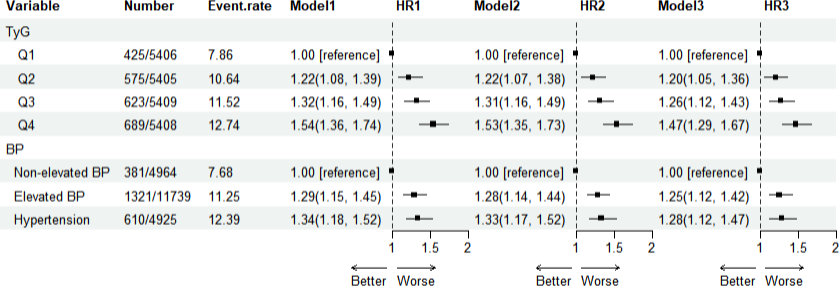
Associations of triglyceride-glucose (TyG) index and blood pressure (BP) with coronary heart disease (CHD). Model 1 was adjusted for age and gender. Model 2 was adjusted for age, gender, classification of disabilities, and grading of disabilities. Model 3 was adjusted for age, gender, classification of disabilities, grading of disabilities, marital status, education level, total cholesterol (TC), serum creatinine (SCr), total protein (TP), and hemoglobin. HR: hazard ratio.

During a median follow-up of 77.45 (IQR 44.81-108.17) months from 2012 to 2022, a total of 2312 participants developed CHD, resulting in an incidence rate of 10.69%. Figure 1 illustrates the association between BP and the TyG index with CHD events. After adjusting for potential confounders in model 3, compared to nonelevated BP, the adjusted HR (aHR) for CHD events in the hypertension group was 1.28 (95% CI 1.12-1.47). When comparing TyG quartile 4 to quartile 1, the aHR for CHD events was 1.47 (95% CI 1.29-1.67).

### Mediation and Interaction Analyses of TyG Between BP and CHD

**Figure 2.**
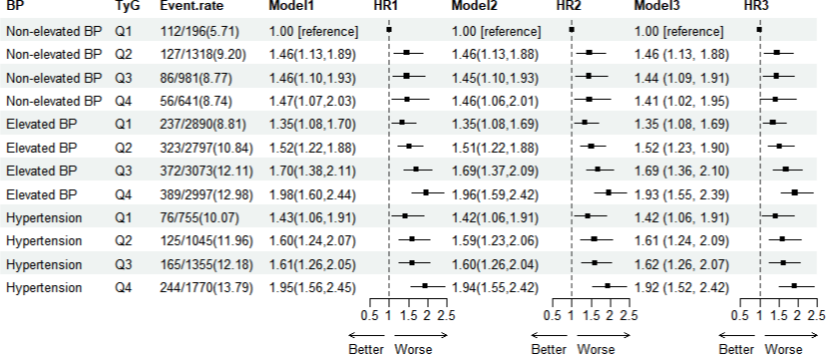
Joint associations of triglyceride-glucose (TyG) index and blood pressure (BP) on coronary heart disease (CHD). Model 1 was adjusted for age and gender. Model 2 was adjusted for age, gender, classification of disabilities, and grading of disabilities. Model 3 was adjusted for age, gender, classification of disabilities, grading of disabilities, marital status, education level, total cholesterol (TC), serum creatinine (SCr), total protein (TP), and hemoglobin. HR: hazard ratio.

The mediation analysis of BP and TyG in relation to the incidence of CHD is shown in Figure S3 in Multimedia Appendix 1 (model 3). The proportion of mediation of BP on CHD incidence through TyG was 20.50% (95% CI 13.60%-22%). Significant multiplicative interactions were observed between TyG and BP on incident CHD (Table 2; multiplicative, HR 1.41, 95% CI 1.02‐1.94). Figure 2 presents the results of the joint analysis of BP and TyG on the outcome. Compared to individuals with nonelevated BP and TyG in the first quartile, those with hypertension and TyG in the fourth quartile had a higher risk of CHD incidence, with an HR of 1.92 (95% CI 1.52‐2.42) after adjusting for confounding factors.

**Table 2.** Interactive effects of triglyceride-glucose index and blood pressure (BP) on coronary heart disease (CHD).

Interactive items	Interactive effects (95% CI)		
	Model 1^a^	Model 2^b^	Model 3^c^
Additive effects			
Relative excess risk due to interaction	1.22 (–0.31 to 2.74)	1.18 (–0.32 to 2.67)	1.01 (–0.36 to 2.39)
Proportion attributable to interaction	0.39 (0.15 to 0.62)	0.38 (0.14 to 0.62)	0.35 (0.10 to 0.60)
Synergy index	2.32 (1.48 to 3.63)	2.29 (1.45 to 3.59)	2.18 (1.36 to 3.50)
Multiplicative effect	1.47 (1.07 to 2.03)	1.46 (1.06 to 2.01)	1.41 (1.02 to 1.94)

a Model 1 was adjusted for age and gender.

b Model 2 was adjusted for age, gender, classification of disabilities, and grading of disabilities.

c Model 3 was adjusted for age, gender, classification of disabilities, grading of disabilities, marital status, education level, total cholesterol (TC), serum creatinine (SCr), total protein (TP), and hemoglobin.

### Group Analysis by Disability Characteristics

**Figure 3.**
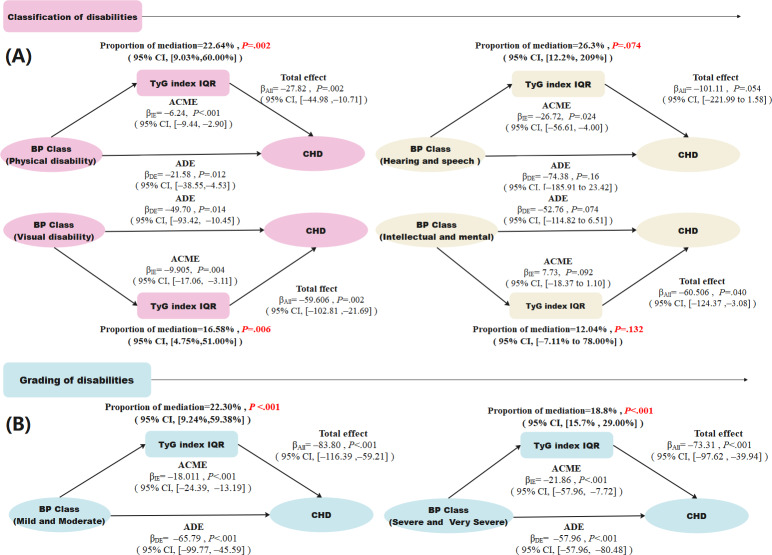
Mediating role of triglyceride-glucose (TyG) index in the association between blood pressure (BP) and coronary heart disease (CHD) according to classification and grading of disability. (A) Mediating role of TyG index in the association between BP and CHD according to the classification of disability and (B) mediating role of the TyG index in the association between BP and CHD according to the grading of disability. All the models were adjusted for age, gender, classification of disabilities, grading of disabilities, marital status, education level, TC, SCr, total protein (TP), and hemoglobin. ACME: average casual mediation effect; ADE: average direct effect; β_DE_: direct effect; β_IE_: indirect effect.

Global interaction tests between disability grading or classification and the combined effects of BP and TyG on CHD showed significant additive and multiplicative interactions after adjustment for covariates (Table 3). Stratified mediation analyses by disability classification (Figure 3A) revealed that the mediation effect of TyG was significant among individuals with physical disabilities (22.64%, 95% CI 9.03%‐60%) and those with visual disabilities (16.58%, 95% CI 4.75%‐51%), whereas the effect was not significant in participants with hearing and speech disabilities (*P*=.074) or intellectual and mental disabilities (*P*=.132). When stratified by disability grading (Figure 3B), the proportion mediated was 22.3% (95% CI 9.24%‐59.38%) in the mild or moderate group and 18.8% (95% CI 15.7%‐29%) in the severe or very severe group.

The joint association analyses (Table S5 in Multimedia Appendix 1) further showed that, compared with individuals in the first quartile of both nonhypertension and TyG, those in the fourth quartile of both hypertension and TyG exhibited a higher risk of CHD across most disability classifications. Specifically, the aHRs were 1.81 (95% CI 1.34‐2.44) for physical disabilities, 1.31 (95% CI 0.77‐2.23) for visual disabilities, 2.81 (95% CI 1.11‐7.09) for hearing and speech disabilities, and 3.51 (95% CI 1.89‐6.50) for intellectual and mental disabilities. Similarly, by disability grading, the HRs were 2.45 (95% CI 1.49‐4.02) for the severe or very severe group and 1.84 (95% CI 1.41‐2.39) for the mild or moderate group.

**Table 3.** Interactive effects of disability classification/grading on blood pressure (BP) and triglyceride-glucose index in relation to coronary heart disease (CHD).

Interactive items	Interactive effects (95% CI)	
	Classification of disabilities	Grading of disabilities
Additive effects		
Relative excess risk due to interaction	0.61 (0.42-1.64)	0.86 (0.27-1.46)
Proportion attributable to interaction	0.41 (0.18-0.98)	0.35 (0.23-0.47)
Synergy index	1.83 (0.88-3.83)	2.45 (2.04-2.93)
Multiplicative effect	1.84 (0.83-1.99)	1.46 (1.27-1.69)

### Subgroup and Sensitivity Analyses

After converting SBP, DBP, and TyG into continuous variables, the mediation analysis was conducted with full adjustment for confounding factors. The results shown in Figure S4 in Multimedia Appendix 1 indicate mediation proportions of 34.5% (*P*<.001) and 34.1% (*P*<.001). The mediation effect of TyG on the association between BP and CHD is greater in participants aged <60 years than in those aged ≥60 years and is more pronounced in male individuals than in female individuals. In the joint analysis, participants in the highest quartile of both hypertension and TyG had an increased risk of CHD compared with those in the lowest quartile. The aHRs were 2.69 (95% CI 1.84‐3.93) in male individuals, 1.50 (95% CI 1.11‐2.02) in female individuals, 2.01 (95% CI 1.59‐2.76) in participants aged <60 years, and 1.58 (95% CI 1.02‐2.44) in those aged ≥60 years.

## Discussion

### Principal Findings

This study expands the understanding of the association between BP, TyG, and CHD by including individuals with disabilities, a group that has often been overlooked in previous research. Our research fills a critical gap by elucidating the significant independent associations of BP and TyG with CHD risk in a cohort of 21,628 individuals with disabilities. Notably, TyG was found to mediate the effect of BP on CHD risk, with this mediation being especially pronounced in individuals with physical disabilities and those with mild or moderate disabilities. Furthermore, significant additive and multiplicative interactions between BP and TyG were observed, with their combination associated with an even higher risk of CHD. These findings suggest that the unique metabolic profiles of these populations may play an important role in their increased CHD risk.

Our results are consistent with existing literature showing that both high BP [24,25] and elevated TyG [26,27] are independent risk factors for CHD. The association between BP and TyG in relation to CVD risk has been studied in normal populations before. One prospective study [17] demonstrated that SBP significantly altered the association between TyG and CVD, with SBP mediating about 10% of the TyG-CVD association, a finding similar to what we observed in our cohort of individuals with disabilities. This suggests that in the population with disabilities, TyG plays a significant role in the pathway linking BP to CHD, possibly reflecting enhanced insulin resistance and metabolic abnormalities in this group. Potential mechanisms for this could include limited physical activity, which often leads to lower metabolic rates and worsened vascular function [28], making TyG a stronger mediator of BP and CHD [29].

A significant synergistic interaction between BP and TyG was identified in our analysis. This result is consistent with prior evidence. A recent Kailuan study [29] also reported a significant interaction between the TyG index and BP status in relation to CVD risk. In addition, a population-based analysis [30] from the National Health and Nutrition Examination Survey found that the combination of a low TyG index and low SBP (<120 mm Hg or <130 mm Hg) was associated with a further reduction in all-cause and cardiovascular mortality, with a particularly strong effect on cardiovascular mortality. Several mechanisms may underlie the interplay between the TyG index and BP in CVD development. Hypertension promotes target organ damage, while an elevated TyG index reflects insulin resistance [31], which drives hyperglycemia, inflammation, oxidative stress, and endothelial dysfunction [32,33]. Together, these factors can accelerate cardiovascular injury and substantially increase CVD risk. Clarifying their joint effects is therefore essential for improving risk assessment and tailoring prevention strategies.

Our findings highlight the need to account for both BP and TyG in CHD risk assessment. The combined assessment of these 2 factors may help identify individuals with disabilities who are at higher risk for CHD. Our study shows that individuals with both elevated BP and TyG have a significantly higher CHD risk compared to those with either factor alone. A few other studies have also reached similar conclusions [17,31]. This suggests a complex, nonlinear association between the two, rather than a simple additive effect. Indeed, prior research has established a significant association between the TyG index and an increased risk of developing hypertension [34-38]. Given this, future risk assessment tools or clinical practices may benefit from integrating BP and TyG management, particularly in individuals with metabolic abnormalities and hypertension, to more effectively manage CHD risk.

Stratified analyses revealed that the mediating role of TyG was particularly strong in physical disabilities, with a moderate effect in visual disabilities, while no significant mediation was detected in hearing and speech or intellectual and mental disabilities. Moreover, the mediation proportion was greater in mild to moderate disabilities than in severe or very severe disabilities. This finding suggests that different types and degrees of disability may amplify the impact of BP on CHD through metabolic pathways. In individuals with physical disabilities or visual disabilities, reduced physical activity and energy expenditure often lead to increased body fat and insulin resistance, which collectively elevate TyG levels [39]. Given the prevalence of metabolic abnormalities in this population, TyG may enhance the impact of BP on CHD risk by reflecting insulin resistance and lipid metabolism disturbances [40]. Furthermore, reduced physical activity may trigger systemic inflammation and endothelial dysfunction, further magnifying the mediating role of TyG. Future research is needed to explore these mechanisms in greater depth and validate the role of TyG in different disability groups. Among individuals with mild to moderate disabilities, the significant mediating effect of TyG may reflect the group’s heightened sensitivity to metabolic stress. These individuals may retain some physiological resilience, but it is limited, making them more vulnerable to the combined impact of elevated BP and higher TyG levels on CHD risk. Furthermore, the relatively preserved health reserves in this group may allow for more precise detection of metabolic effects, which might be overshadowed by other health factors in those with severe disabilities. This highlights the need for targeted interventions to address metabolic health in individuals with disabilities, particularly those with physical disabilities or mild to moderate severity, who may be more susceptible to the metabolic factors that contribute to CHD.

### Strengths and Limitations

The findings of this study have important implications. First, the large sample size of 21,628 individuals with disabilities provides high statistical power and generalizability. Second, this study enhances our understanding of the contribution of metabolic factors to cardiovascular risk in populations with disabilities and addresses a critical gap in the existing literature. Third, by using a novel analytical approach that decomposes the total association between BP, TyG, and CHD into direct and indirect effects, we offer a deeper understanding of the critical role of metabolic factors in cardiovascular health [41]. The stratified analyses further enhance the precision of our findings and provide valuable insights for developing targeted interventions.

However, there are several limitations to this study. First, although the study benefits from a large sample size, its retrospective cohort design inherently limits causal inference, restricting the ability to draw definitive conclusions about the temporal association between exposures and outcomes. In addition, we recognize that factors such as lifestyle behaviors, health care access, and socioeconomic status are important, but due to current data limitations, we have adjusted for other objective health indicators in an attempt to minimize their potential impact. Additionally, the uniform access to living support provided by the Shanghai Disability Federation for all participants may help reduce the influence of these unmeasured factors. Third, since all data were collected from Shanghai, the generalizability of the findings to other regions or countries may be limited, and future studies will aim to expand the data by incorporating additional variables and conducting multi-center prospective cohort studies to validate these results in a broader context.

### Conclusions

In conclusion, we identified a significant association between BP and the TyG index with CHD risk in individuals with disabilities, with TyG acting as a prominent mediator in the association between BP and CHD. This mediation effect was particularly pronounced in individuals with physical disabilities and those classified with mild-to-moderate disability severity. Our findings underscore the importance of addressing the combined effects of insulin resistance and blood pressure in high-risk populations. Integrated intervention strategies targeting both factors may be crucial for reducing CHD risk.

## Supplementary material

10.2196/78068 Multimedia Appendix 1 Multimedia appendix. Additional tables and figures supporting the main results.

## Abbreviations

aHR: adjusted hazard ratio
BP: blood pressure
CHD: coronary heart disease
CVD: cardiovascular disease
DBP: diastolic blood pressure
HR: hazard ratio
NIE: natural indirect effect
RCS: restricted cubic spline
SBP: systolic blood pressure
TyG: triglyceride-glucose
