# Supplementary material for: Association Between Triglyceride-Glucose Index, Blood Pressure Status, and Coronary Heart Disease Risk Among Chinese Adults With Disabilities: 10-Year Disability Health Survey Cohort Study
Source: JMIR Public Health Surveill. 2025 Nov 3;11:e78068. doi: 10.2196/78068 (PMC12975414; doi:10.2196/78068)
Supplement: Multimedia Appendix 1 [file publichealth-v11-e78068-s001.docx]

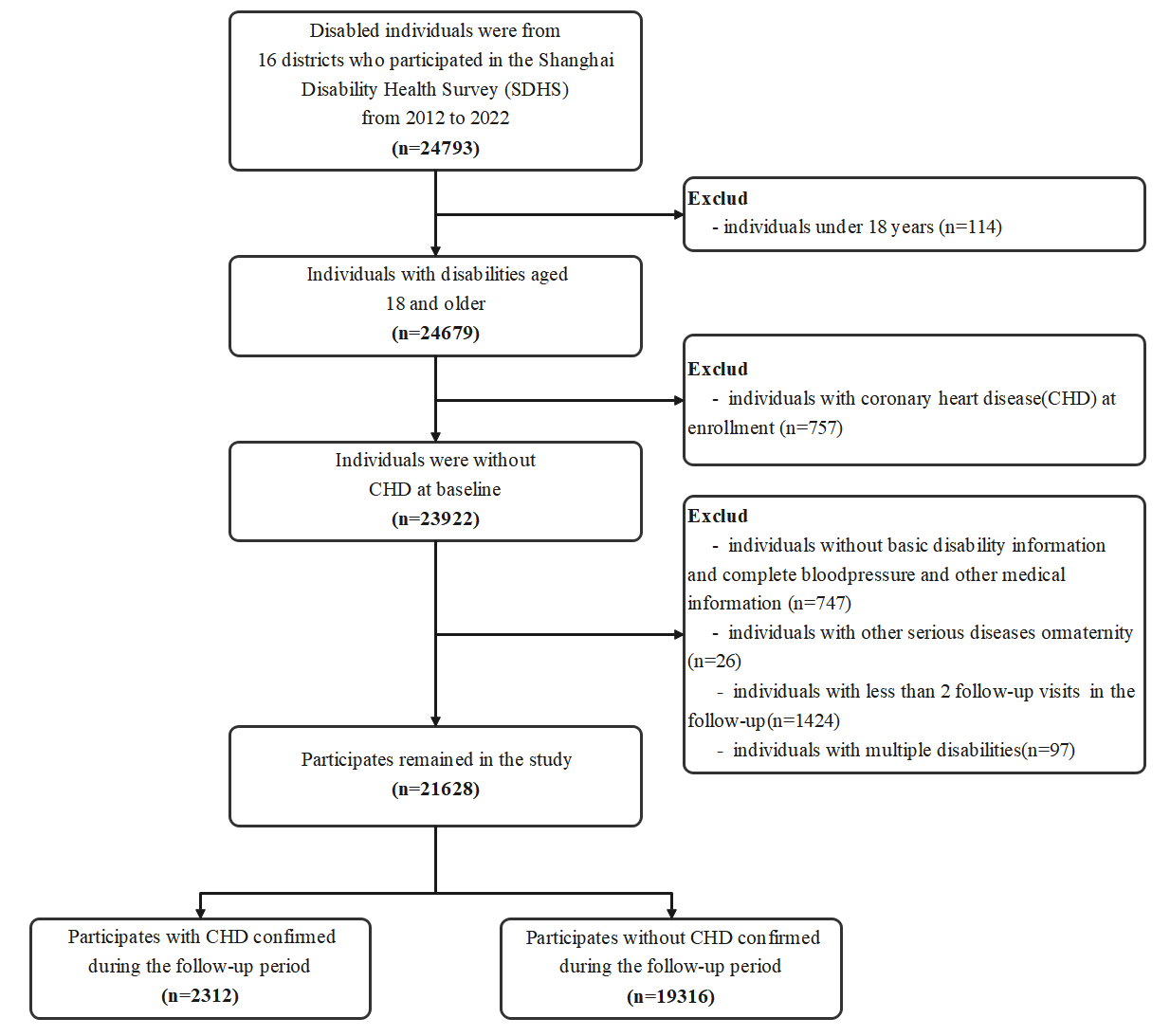


Figure S1. Consort flow diagram for participants included in the study

TableS1. Missing Data Summary for Each Variable

| **Variable** | **Missing Count** | **Missing Proportion (%)** |
| --- | --- | --- |
| **BMI , kg/m2** | 1345 | 6.22 |
| Marital status (n, %) | 512 | 2.37 |
| Education | 723 | 3.34 |
| FBG, mmol/L | 184 | 0.85 |
| TC, mmol/L | 299 | 1.38 |
| TG, mmol/ | 298 | 1.38 |
| TP, g/L | 482 | 2.23 |
| Alb, g/L | 256 | 1.18 |
| Glo, g/L | 410 | 1.9 |
| Alt, U/L | 556 | 2.57 |
| UA, μmol/L | 233 | 1.08 |
| SCr, μmol/L | 198 | 0.92 |
| SU, mmol/L | 177 | 0.82 |
| Hb, g/L | 134 | 0.62 |
| PLT, 10^9/L | 163 | 0.75 |

BMI body mass index, FBG fasting plasma glucose, TC total cholesterol, TG total triglyceride, TP total protein, Alb albumin, Glo globulin, Alt alanine aminotransferase, UA uric acid, SCr serum creatinine, SU serum urea, Hb hemoglobin, PLT platelet count

TableS2. Incidence Rate Per Year.

| **Year** | **Number of Cases** | **Incidence Rate (%)** |
| --- | --- | --- |
| 2012 | 15 | 0.65 |
| 2013 | 141 | 6.09 |
| 2014 | 345 | 14.91 |
| 2015 | 219 | 9.47 |
| 2016 | 263 | 11.37 |
| 2017 | 236 | 10.21 |
| 2018 | 242 | 10.46 |
| 2019 | 179 | 7.73 |
| 2020 | 152 | 6.57 |
| 2021 | 295 | 12.77 |
| 2022 | 225 | 9.72 |

TableS3. Baseline characteristics of 21,628disabilities by triglyceride-glucose index quartiles.

| Variables | | Total sample  (n=21628) | Q1  (5.20, 8.19] | Q2  (8.19, 8.5] | Q3  (8.56, 8.97] | Q4  (8.97,12.4] | *P* |
| --- | --- | --- | --- | --- | --- | --- | --- |
| **Age, years** | 53.30±10.57 | | 51.10±11.70 | 53.40±10.70 | 54.5±9.93 | 54.3±7.38 | <0.001 |
| **Gender, n (%)** | | |  |  |  |  | <0.001 |
| Male | 11007(50.89) | | 2715(50.22) | 2639(48.80) | 2628(48.6) | 3025(56.00) |  |
| Female | 10621(49.11) | | 2691(49.78) | 2766 (51.20) | 2781(51.4) | 2383(44.00) |  |
| **BMI , kg/m2** | 24.09±3.58 | | 22..20±3.12 | 23.60±3.37 | 24.80±3.45 | 25.70±3.41 | <0.001 |
| **Marry state, n (%)** | | |  |  |  |  | <0.001 |
| Married | 17852(82.54) | | 4263(78.90) | 4464(82.60) | 4584(84.70) | 4541 (84.00) |  |
| Other | 3776 (14.46) | | 1143(21.10) | 941(17.40) | 825(15.30) | 867(16.00) |  |
| **Education** |  | |  |  |  |  | 0.137 |
| Primary school & Illiterate | 4683(21.7) | | 1197 (22.1) | 1145(21.2) | 1110(20.5) | 1231 (22.8) |  |
| Junior high school | 11199 (51.8) | | 2811(52.0) | 2821(52.5) | 2838(52.5) | 1231(22.8) |  |
| Senior high school | 4896 (22.6) | | 1200(22.2) | 1235 (22.8) | 1230(22.7) | 385(69.4) |  |
| College & Higher | 850 (3.93) | | 198(3.66) | 204(3.77) | 231(4.27) | 217(4.01) |  |
| **Classification of disabilities, n (%)** | | |  |  |  |  | <0.001 |
| Intellectual & mental disability | 3799 (17.6) | | 1102 (20.4) | 936(17.3) | 860(15.9) | 901(16.7) |  |
| Hearing & speech disability | 1775 (8.21) | | 492(9.10) | 462(8.55) | 416(7.69) | 405(7.49) |  |
| Visual disability | 4153 (19.2) | | 985(18.2) | 1035(19.1) | 1053(19.5) | 1080(20.0) |  |
| Physical disability | 11901 (55.0) | | 2827(52.3) | 2972(55l.0) | 3080(56.9) | 3022(55.9) |  |
| **Grading of disabilities, n (%)** | | |  |  |  |  | 0.447 |
| Very severe disability | 1924 (8.90) | | 516(9.54)2 | 459(8.49) | 462(8.54) | 487(9.01) |  |
| Severe disability | 2729 (12.6) | | 669(12.4) | 674(12.5) | 693(12.8) | 693(12.8) |  |
| Moderate disability | 7458(34.5) | | 1851(34.2) | 1878(34.7) | 1828(33.8) | 1901(35.2) |  |
| Mild disability | 9517(44.0) | | 370(43.8) | 1035(19.1) | 2426 (44.9) | 2327(43.0) |  |
| **Comorbidities, n (%)** | | |  |  |  |  |  |
| Hypertension | 5961(27.6) | | 796(14.7) | 1245(23.0) | 1706(31.5) | 2214(40.9) | <0.001 |
| Hyperlipemia | 1317(6.09) | | 90(1.66) | 162(3.00) | 298(5.51) | 767(14.2) | <0.001 |
| **Blood pressure** |  | |  |  |  |  |  |
| SBP | 135.15±20.87 | | 127.56 ± 19.87 | 133.17 ± 20.05 | 137.55 ± 20.25 | 142.32 ± 20.42 | <0.001 |
| DBP | 79.31±12.46 | | 74.75 ± 12.16 | 78.10 ± 12.02 | 80.61 ± 11.98 | 83.79 ± 11.88 | <0.001 |
| **Metabolic Biomarkers** | | |  |  |  |  |  |
| FBG, mmol/L | 5.60±1.53 | | 5.02 ± 0.51 | 5.26 ± 0.68 | 5.51 ± 0.97 | 6.59 ± 2.51 | <0.001 |
| TC, mmol/L | 4.37 ± 0.77 | | 4.70 ± 0.83 | 4.91 ± 0.87 | 5.14 ± 0.96 | 4.37 ± 0.77 | <0.001 |
| TG,mmol/ | 1.22(0.8,1.75) | | 0.7(0.61,0.81) | 1.05(0.96,1.16) | 1.47(1.32,1.65) | 2.31(1.94,2.95) | <0.001 |
| TP, g/L | 72.38±3.61 | | 71.21 ± 4.09 | 72.05 ± 4.04 | 72.70 ± 3.95 | 73.56 ± 4.33 | <0.001 |
| Alb, g/L | 43.43±2.33 | | 43.05 ± 2.41 | 43.30 ± 2.36 | 43.54 ± 2.24 | 43.84 ± 2.27 | <0.001 |
| Glo, g/L | 28.97±3.61 | | 28.30 ± 3.61 | 28.80 ± 3.50 | 29.14 ± 3.46 | 29.63 ± 3.72 | <0.001 |
| Alt, U/L | 20.00  (15.00,29.00) | | 17.00  (13.00,23.00) | 19.00  (14.00,26.00) | 21.00  (16.00,29.00) | 25.00  (18.00,37.00) | <0.001 |
| UA, μmol/L | 318.40±87.54 | | 286.14 ± 75.97 | 308.75 ± 80.86 | 324.62 ± 84.66 | 354.07 ± 93.43 | 0.943 |
| SCr, μmol/L | 62.10  (50.70, 73.32) | | 60.10  (49.30,71.00) | 61.90  (50.60,72.90) | 62.80  (51.50,74.00) | 63.60  (51.70,75.50) | <0.001 |
| SU,mmol/L | 5.00(4.20,6.00) | | 5.10(4.30,6.00) | 5.00(4.20,5.90) | 5.00(4.20,5.90) | 5.00(4.30,6.00) | <0.001 |
| Hb, g/L | 137.01±15.27 | | 133.73 ± 15.29 | 135.95 ± 14.99 | 137.54 ± 14.85 | 140.85 ± 15.08 | <0.001 |
| PLT, 109/L | 200.88±58.27 | | 200.31 ± 58.30 | 203.73 ± 58.98 | 202.96 ± 57.94 | 200.31 ± 58.30 | <0.001 |

Data presented as number (%) or mean±SD (for these with normal distribution).

BMI body mass index, FBG fasting plasma glucose, TC total cholesterol, TG total triglyceride, TP total protein, Alb albumin, Glo globulin, Alt alanine aminotransferase, UA uric acid, SCr serum creatinine, SU serum urea, Hb hemoglobin, PLT platelet count

^a^ The P value was determined using the Chi-square test for categorical data, ANOVA for continuous data across categorical groups, and the Kruskal-Wallis test for continuous data that is ordinal or non-normally distributed.

^b^ The TyG was calculated by the formula ln[TC (mg/dl) × FBG (mg/dl) / 2]


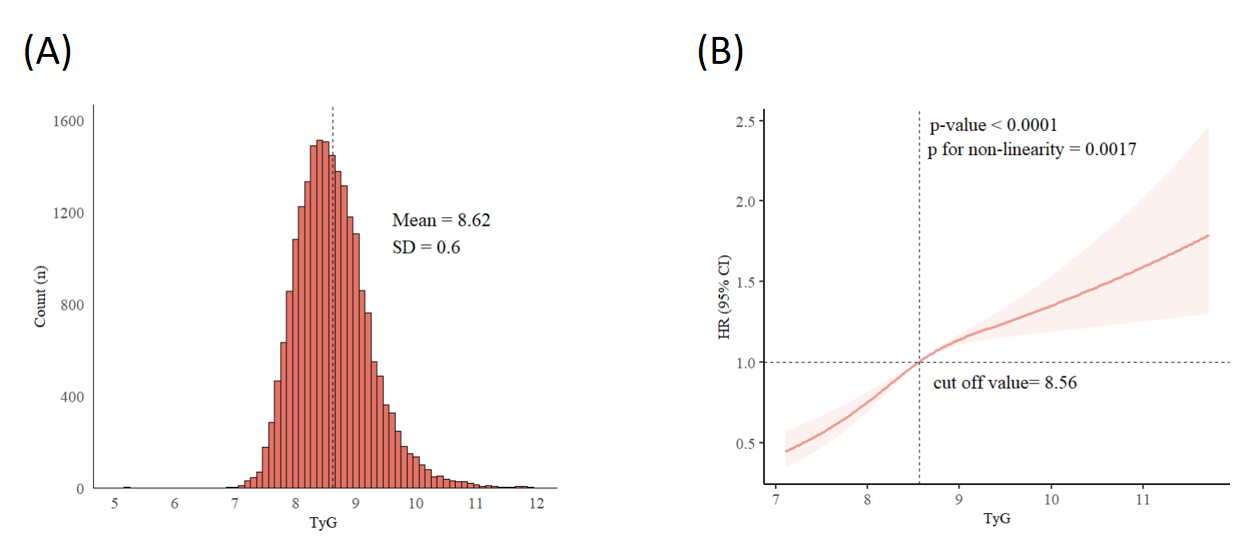


Figure S2. Dose-responsive relationship of the Triglyceride-Glucose Indexand Coronary Heart Disease. The A graphs illustrates the distribution of the Triglyceride-Glucose Index. The B graphs show HRs for Coronary Heart Disease adjusted for age, gender, classification of disabilities , grading of disabilities, marital status, education level, TC , SCr, TP, Hb.

TableS4. Baseline characteristics of 21,628disabilities by triglyceride-glucose index quartiles.

| **Model** | **Variable** | **Chi-squared** | **df** | **p-value** |
| --- | --- | --- | --- | --- |
| **Blood Pressure Class and CHD** | |  |  |  |
| **Model 1** | **GLOBAL** | **14.041** | **5** | **0.0154** |
|  | TyG index IQR | 2.214 | 3 | 0.5291 |
|  | Age | 11.242 | 1 | 0.0008 |
|  | Sex | 3.44 | 3 | 0.329 |
| **Model 2** | **GLOBAL** | **15.477** | **8** | **0.0505** |
|  | TyG index IQR | 1.642 | 3 | 0.6498 |
|  | Sex | 3.43 | 3 | 0.327 |
|  | Disability Level | 0.497 | 1 | 0.4809 |
|  | Disability Category | 3.654 | 3 | 0.3013 |
| **Model 3** | **GLOBAL** | **22.614** | **14** | **0.0669** |
|  | TyG index IQR | 2.352 | 3 | 0.5027 |
|  | Disability Level | 0.497 | 1 | 0.4809 |
|  | Disability Category | 3.654 | 3 | 0.3013 |
|  | Sex | 0.438 | 1 | 0.5082 |
|  | Age | 10.177 | 1 | 0.0014 |
|  | TC | 3.294 | 1 | 0.0695 |
|  | SCr | 0.703 | 1 | 0.4016 |
|  | TP | 2.807 | 1 | 0.0939 |
|  | Hb | 0.338 | 1 | 0.5609 |
| **TyG index IQR and CHD** | |  |  |  |
| **Model 1** | **GLOBAL** | **15.854** | **4** | **0.00322** |
|  | BP Class | 2.563 | 2 | 0.27759 |
|  | Sex | 0.361 | 1 | 0.5478 |
|  | Age | 11.044 | 1 | 0.00089 |
| **Model 2** | GLOBAL | 17.422 | 8 | 0.026 |
|  | BP Class | 1.735 | 2 | 0.42 |
|  | Disability Level | 0.015 | 1 | 0.90 |
|  | Disability Category | 5.657 | 3 | 0.13 |
|  | Age | 10.279 | 1 | 0.0013 |
|  | Sex | 0.437 | 1 | 0.51 |
| **Model 3** | **GLOBAL** | 22.752 | 13 | 0.0448 |
|  | BP Class | 2.61 | 2 | 0.2711 |
|  | Disability Level | 0.596 | 1 | 0.4403 |
|  | Disability Category | 3.494 | 3 | 0.3215 |
|  | Sex | 0.41 | 1 | 0.5222 |
|  | Age | 10.009 | 1 | 0.0016 |
|  | TC | 3.262 | 1 | 0.0709 |
|  | SCr | 0.92 | 1 | 0.3375 |
|  | TP | 2.987 | 1 | 0.084 |
|  | Hb | 0.322 | 1 | 0.5851 |
|  | PLT | 0.842 | 1 | 0.3589 |


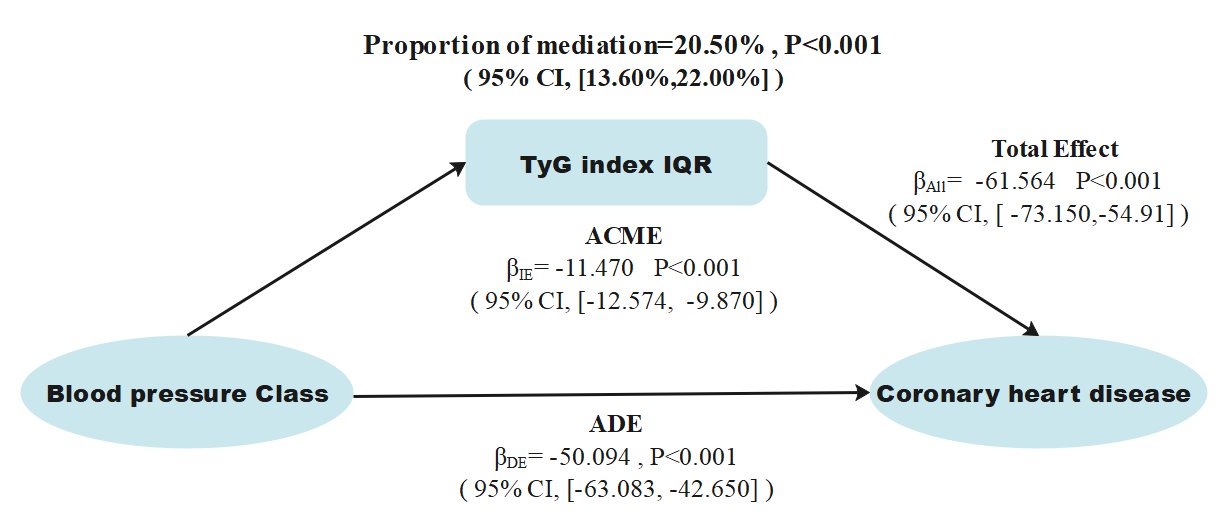


FigureS3 The mediating effect of triglyceride-glucose index on the relationship between BP and CHD; β_IE_，Indirect effect；β_DE,_ Direct effect；ADE，Average Direct Effect；ACME，Average Causal Mediation Effect;

Model was adjusted for a age, gender, classification of disabilities , grading of disabilities, marital status, education level,TC , SCr, TP, Hb

TableS5. Interactive role of triglyceride-glucose index in the relationship between BP and CHD according to classification and grading of disability

| BP | TyG | | HR (95%CI) | | | |  | |
| --- | --- | --- | --- | --- | --- | --- | --- | --- |
|  |  |  | Physical disabilities a | Visual disabilitya | Hearing & speech disability**^a^** | Intellectual & mental disability**^a^** | Severe&Very severe **^a^** | Mild& Moderate **^a^** |
| **Non-elevated BP** | | Q1 | ref | ref | ref | ref | ref | ref |
| Non-elevated BP | | Q2 | 1.17 (0.83, 1.66) | 1.80 (1.06, 3.07) | 2.11 (0.85, 5.28) | 1.93 (1.00, 3.74) | 1.65 (0.97, 2.83) | 1.43 (1.07, 1.90) |
| Non-elevated BP | | Q3 | 1.60 (1.12, 2.28) | 1.22 (0.64, 2.32) | 1.33 (0.46, 3.88) | 0.87 (0.34, 2.23) | 0.89 (0.45, 1.78) | 1.61 (1.18, 2.20) |
| Non-elevated BP | | Q4 | 1.26 (0.82, 1.96) | 1.15 (0.56, 2.38) | 2.55 (0.81, 8.03) | 1.93 (0.86, 4.29) | 2.07 (1.10, 3.89) | 1.27 (0.87, 1.86) |
| **Elevated BP** | | Q1 | 1.22 (0.91, 1.64) | 1.31 (0.81, 2.15) | 1.58 (0.67, 3.74) | 1.77 (0.98, 3.22) | 1.57 (0.97, 2.55) | 1.32 (1.02, 1.71) |
| Elevated BP | | Q2 | 1.52 (1.15, 2.02) | 1.21 (0.74, 1.96) | 2.20 (0.98, 4.93) | 1.61 (0.87, 3.00) | 1.61 (1.01, 2.59) | 1.53 (1.19, 1.95) |
| Elevated BP | | Q3 | 1.50 (1.13, 1.99) | 1.92 (1.21, 3.06) | 1.69 (0.71, 4.04) | 1.88 (1.03, 3.42) | 2.01 (1.26, 3.19) | 1.65 (1.29, 2.11) |
| Elevated BP | | Q4 | 1.76 (1.33, 2.34) | 2.03 (1.27, 3.24) | 3.01 (1.32, 6.87) | 1.69 (0.91, 3.14) | 2.16 (1.36, 3.43) | 1.90 (1.49, 2.43) |
| **Hypertension** | | Q1 | 1.33 (0.91, 1.94) | 1.28 (0.67, 2.47) | 0.97 (0.25, 3.72) | 2.25 (1.03, 4.90) | 1.24 (0.62, 2.47) | 1.46 (1.06, 2.03) |
| Hypertension | | Q2 | 1.38 (0.98, 1.95) | 1.86 (1.08, 3.20) | 2.33 (0.88, 6.12) | 1.67 (0.78, 3.58) | 2.28 (1.33, 3.88) | 1.47 (1.09, 1.98) |
| Hypertension | | Q3 | 1.45 (1.05, 1.99) | 1.21 (0.69, 2.13) | 2.98 (1.20, 7.37) | 2.71 (1.39, 5.32) | 1.78 (1.04, 3.04) | 1.59 (1.20, 2.10) |
| Hypertension | | Q4 | 1.81 (1.34, 2.44) | 1.31 (0.77, 2.23) | 2.81 (1.11, 7.09) | 3.51 (1.89, 6.50) | 2.45 (1.49, 4.02) | 1.84 (1.41, 2.39) |

Model was adjusted for age, gender, classification of disabilities , grading of disabilities, marital status, education level,,TC , SCr, TP, Hb


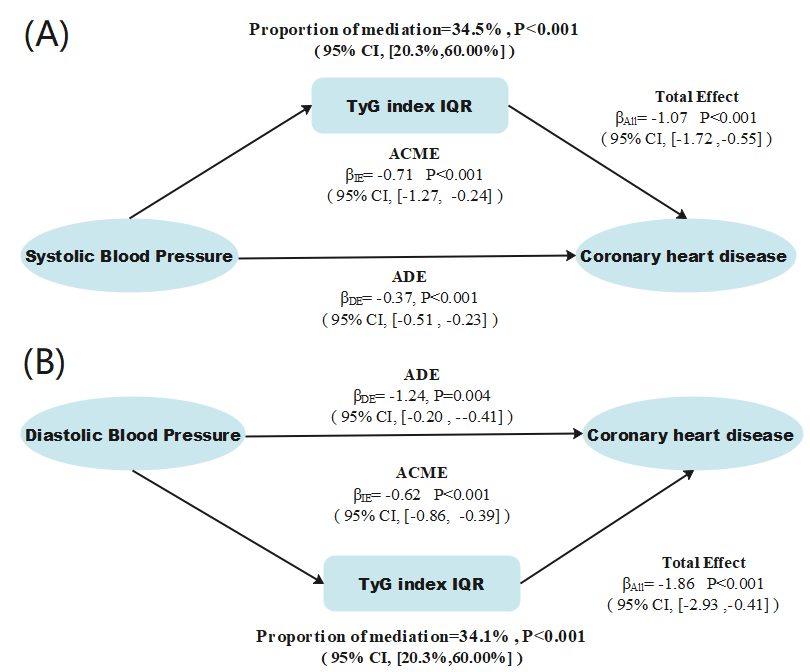


Figure S4. The mediating effect of triglyceride-glucose index and Continuous blood pressure variables on CHD. A, the mediating effect of TyG on the relationship between systolic blood pressure and CHD; B, the mediating effect of TyG on the relationship between diastolic blood pressure and CHD.

β_IE_，Indirect effect；β_DE_，Direct effect；ADE，Average Direct Effect；ACME，Average Causal Mediation Effect


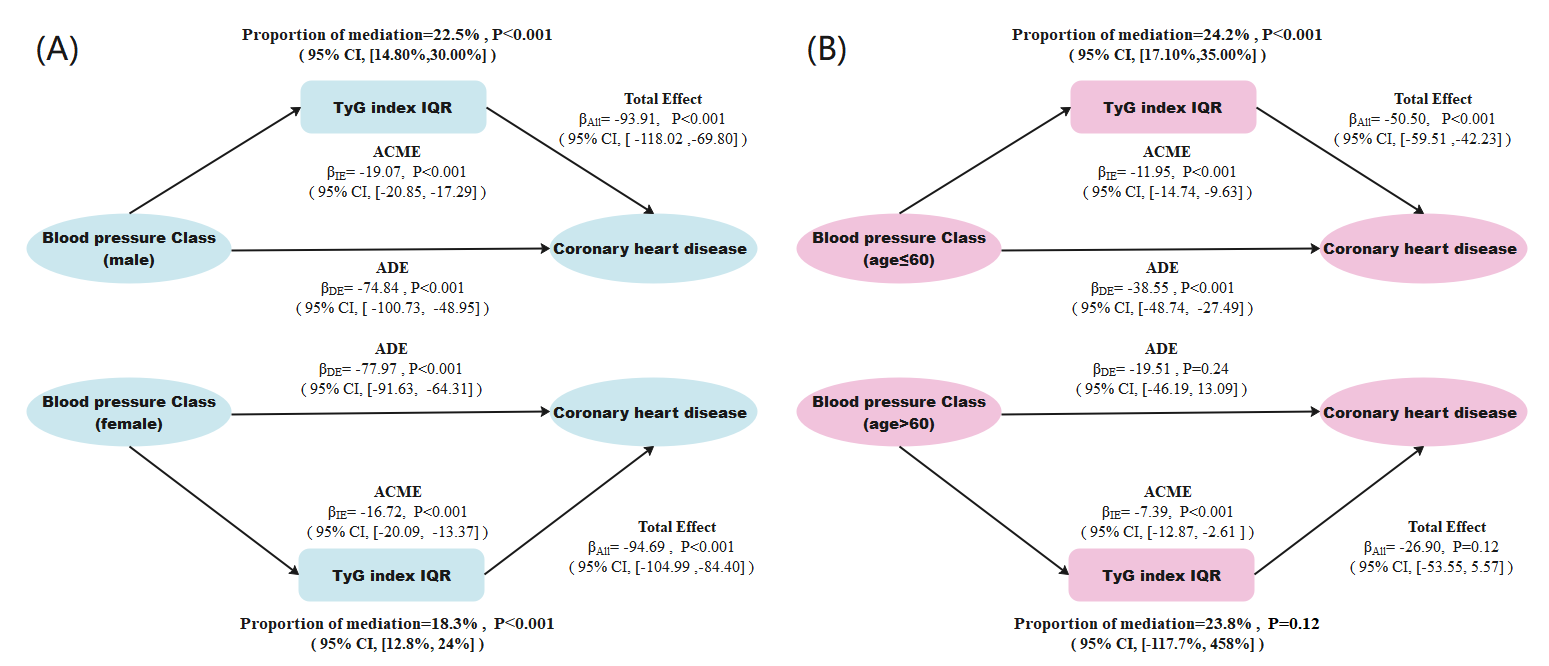


Figure S5. The mediating effect of triglyceride-glucose index and blood pressure class on CHD. A, the mediating effect of triglyceride-glucose index and blood pressure class on CHD according to sex; B, the mediating effect of triglyceride-glucose index and blood pressure class on CHD according to age.

β_IE_，Indirect effect；β_DE_，Direct effect；ADE，Average Direct Effect；ACME，Average Causal Mediation Effect

Table S6 Joint associations of triglyceride-glucose index and BP with CHD analyzed by gender.

| BP | TyG |  | |  | HR (95%CI) | | | |
| --- | --- | --- | --- | --- | --- | --- | --- | --- |
|  |  |  |  | **Female**  (n=10621) | | **Male**  (n=11007) |  | |
| **Non-elevated BP** | | Q1 |  | ref | | ref | |  |
| Non-elevated BP | | Q2 |  | 1.29 (0.94, 1.77) | | 1.75 (1.14, 2.70) | |  |
| Non-elevated BP | | Q3 |  | 1.20 (0.84, 1.72) | | 1.87 (1.18, 2.97) | |  |
| Non-elevated BP | | Q4 |  | 1.24 (0.81, 1.90) | | 1.82 (1.10, 3.02) | |  |
| **Elevated BP** | | Q1 |  | 1.25 (0.94, 1.66) | | 1.60 (1.10, 2.34) | |  |
| Elevated BP | | Q2 |  | 1.32 (1.01, 1.73) | | 1.86 (1.29, 2.69) | |  |
| Elevated BP | | Q3 |  | 1.36 (1.04, 1.79) | | 2.28 (1.59, 3.28) | |  |
| Elevated BP | | Q4 |  | 1.56 (1.18, 2.05) | | 2.63 (1.84, 3.78) | |  |
| **Hypertension** | | Q1 |  | 1.41 (0.97, 2.05) | | 1.53 (0.95, 2.46) | |  |
| Hypertension | | Q2 |  | 1.45 (1.05, 2.00) | | 1.78 (1.14, 2.76) | |  |
| Hypertension | | Q3 |  | 1.37 (1.01, 1.87) | | 1.96 (1.30, 2.95) | |  |
| Hypertension | | Q4 |  | 1.50 (1.11, 2.02) | | 2.69 (1.84, 3.93) | |  |

Model was adjusted age, gender, classification of disabilities , grading of disabilities, marital status, education level,,TC , SCr, TP, Hb

Table S7 Joint associations of triglyceride-glucose index and BP with CHD analyzed by age.

| BP | TyG | |  | | |  | HR (95%CI) | | | | |
| --- | --- | --- | --- | --- | --- | --- | --- | --- | --- | --- | --- |
|  |  |  |  |  |  | **Age≤60**  (n=16144) | | **Age>60**  (n=5484) | |  | |
| **Non-elevated BP** | | Q1 | |  | ref | | | | ref | |  |
| Non-elevated BP | | Q2 | |  | 1.47 (1.09, 1.98) | | | | 1.46 (0.89, 2.39) | |  |
| Non-elevated BP | | Q3 | |  | 1.47 (1.06, 2.03) | | | | 1.25 (0.70, 2.24) | |  |
| Non-elevated BP | | Q4 | |  | 1.49 (1.04, 2.16) | | | | 1.16 (0.58, 2.34) | |  |
| **Elevated BP** | | Q1 | |  | 1.38 (1.05, 1.80) | | | | 1.28 (0.84, 1.95) | |  |
| Elevated BP | | Q2 | |  | 1.66 (1.29, 2.15) | | | | 1.29 (0.85, 1.94) | |  |
| Elevated BP | | Q3 | |  | 1.81 (1.40, 2.34) | | | | 1.45 (0.97, 2.18) | |  |
| Elevated BP | | Q4 | |  | 1.99 (1.54, 2.57) | | | | 1.75 (1.16, 2.64) | |  |
| **Hypertension** | | Q1 | |  | 1.75 (1.22, 2.50) | | | | 1.03 (0.61, 1.74) | |  |
| Hypertension | | Q2 | |  | 1.50 (1.08, 2.09) | | | | 1.64 (1.05, 2.56) | |  |
| Hypertension | | Q3 | |  | 1.35 (0.99, 1.86) | | | | 1.82 (1.18, 2.79) | |  |
| Hypertension | | Q4 | |  | 2.10 (1.59, 2.76) | | | | 1.58 (1.02, 2.44) | |  |

Model was adjusted for age, gender, classification of disabilities , grading of disabilities, marital status, education level,,TC , SCr, TP, Hb
